# Preservation of residual hearing after cochlear implant surgery with slim modiolar electrode

**DOI:** 10.1007/s00405-019-05708-x

**Published:** 2019-10-31

**Authors:** Matti Iso-Mustajärvi, Sini Sipari, Heikki Löppönen, Aarno Dietz

**Affiliations:** 1 University of Eastern Finland, Yliopistonranta 1, 70210 Kuopio, Finland; 2 Kuopio University Hospital, Ear, Nose and Throat Diseases, KNK-klinikka, Puijonlaaksontie 2, 70210 Kuopio, Finland

**Keywords:** Cochlear implant, Hearing preservation, Modiolar electrode, Electro-acoustic stimulation

## Abstract

**Purpose:**

To evaluate the insertion results and hearing preservation of a novel slim modiolar electrode (SME) in patients with residual hearing.

**Methods:**

We retrospectively collected the data from the medical files of 17 patients (18 ears) implanted with a SME. All patients had functional low frequency hearing (PTA _(0.125–0.5 kHz)_ ≤ 80 dB HL). The insertion results were re-examined from the postoperative cone-beam computed tomography scans. Postoperative thresholds were obtained at the time of switch-on of the sound processors (mean 43 days) and at latest follow-up (mean 582 days). The speech recognition in noise was measured with the Finnish matrix sentence test preoperatively and at follow-up.

**Results:**

The mean insertion depth angle (IDA) was 395°. Neither scala dislocations nor tip fold over were detected. There were no total hearing losses. Functional low-frequency hearing was preserved in 15/18 (83%) ears at switch-on and in 14/17 (82%) ears at follow-up. According to HEARRING classification, 55% (10/18) had complete HP at switch-on and 41% (7/17) still at follow-up. Thirteen patients (14 ears) were initially fitted with electric–acoustic stimulation and seven patients (8 ears) continued to use it after follow-up.

**Conclusions:**

The preliminary hearing preservation results with the SME were more favorable than reported for other perimodiolar electrodes. The results show that the array may also be feasible for electro-acoustic stimulation; it is beneficial in that it provides adequate cochlear coverage for pure electrical stimulation in the event of postoperative or progressive hearing loss.

## Introduction

The preservation of the delicate inner ear structures has become a major consideration in cochlear implant surgery as intracochlear trauma has been shown to negatively affect the post-implant hearing results [1–5]. Due to the more advanced surgical techniques and more delicate electrode arrays, post-operative results have improved during recent years. This has led to an expansion of the use of these devices, now including also patients with functional residual hearing. Patients with substantial residual hearing in the lower frequencies may benefit from cochlear implantation by combined electric–acoustic stimulation (EAS), provided that their hearing can be preserved at surgery. First described by von Ilberg et al. [6], the physiological acoustic stimulation in the low frequencies combined with the electric stimulation by the cochlear implant has been shown to enhance the post-operative hearing results in terms of better sound quality, improved music listening abilities and better speech recognition against background noise [7–11] Although residual hearing can be preserved with longer lateral wall electrodes (LWE), much more favorable results have been reported for shorter LWE. For short electrodes (≤ 20 mm active length), the hearing preservation rates vary from 54 to 88%, depending on the classification [10–14]. The disadvantage of short arrays is that in the event of a total postoperative hearing loss, the incomplete cochlear coverage may compromise the outcome with pure electrical stimulation. For electric hearing, deeper insertion angles have been shown to provide significantly better speech perception results [4, 15, 16]. The hearing preservation results for these standard length LWEs (i.e. > 20 mm active length) vary from 11.3 to 77.7% [14, 17–20].

Conventional (i.e. stylet type) perimodiolar electrodes (PME) are reported to cause more trauma as compared to lateral wall electrodes (LWE) [16, 21, 22, 23]. Thus, the use of PMEs for hearing preservation surgery is seldom justified. Due to the closer proximity to the modiolus and the spiral neurons, PMEs may provide electro-physiological advantages, such as lower current consumptions and possibly more localized stimulation. However, there are no convincing data that these potential benefits are related to better clinical outcomes [3, 24–26].

A new PME, the slim modiolar electrode (SME) (Cochlear Company, Sydney, Australia) was recently designed for atraumatic insertion. The aim of this study was to analyze the clinical insertion and hearing preservation results of the SME.

## Materials and methods

We retrospectively collected the data from the medical files of 17 patients (18 ears) implanted with the SME. Patients with relevant functional hearing, defined as preoperative low-frequency PTA _(0.125–0.5 kHz_) ≤ 80 dB (HL) were included in this study [27]. Patients with vestibulo-cochlear anomalies or cochlear fibrosis and/or ossification were excluded. The study had institutional approval (No. 5551850). Preoperative hearing thresholds were available from all patients and results from the Finnish Matrix Sentence Test (FMST) in 16 patients. Speech recognition was measured with the novel FMST, the standard speech-in-noise test was used in adult CI recipients to measure hearing performance [28, 29]. Randomized 20-sentence test lists and a non-fluctuating speech-spectrum shaped noise at a constant level of 65 dB SPL were used as speech and noise signals. The speech reception threshold (SRT), i.e. the signal-to-noise ratio at which 50% of the test items are correctly recognized, was determined in an adaptive test measurement procedure. One child was an immigrant with insufficient language skills and the other child had mild autism spectrum disorder (cases 4 and 7) and could not perform the FMST. All measurements were performed in the best-aided condition.

All patients underwent routine pre-operative magnetic resonance imaging (MRI) and high-resolution computed tomography (HRCT) to rule out cochlear malformation or retrocochlear pathology. All patients had normal temporal bone and labyrinthine anatomy.

The SME is a new generation PME, whose volume is approximately 40% smaller than the Contour array. The internal stylet was replaced by an external sheath to keep the electrode straight prior to its insertion. The SME has a diameter of 0.35 × 0.4 mm at the tip and 0.45 mm × 0.5 mm at the base.

All patients underwent cochlear implantation via a trans-mastoid posterior tympanotomy approach under general anesthesia according to the institution’s hearing preservation protocol. The patients were given cefuroxime 1.5 g and dexamethasone 7.5–10 mg intravenously during induction. Weight equivalent doses were administered to the pediatric patients. The bony overhang over the round window (RW) was carefully drilled down to largely expose the round window membrane (RWM). A Spongostan (Ferrosan, Copenhagen, Denmark) soaked with dexamethasone 10 mg/ml was placed into the RWM for the time of implant bed drilling. The RWM was incised in the anterior part and lifted posteriorly with a short hook to open the anterior half of the round window. A hyaluronic acid–dexamethasone mixture (50:50 ratio) was then applied onto the RW area. Prior to loading the electrode, the hyaluronic acid–dexamethasone mixture was applied onto the array to ensure smooth gliding of the sheath during insertion. During insertion, special attention was paid to the appropriate orientation of the wing. The insertion and the removal of the sheath were performed as slowly as possible. The final position of the array was finally adjusted, with the distal marker inside the cochlea and the proximal marker outside. The white triangle was locked between the chorda-facial angle and was secured with bone paste and fibrin glue for stabilization. Finally, a tiny piece of temporal fascia was prepared and placed around the array to seal the RW.

**Fig. 1:**
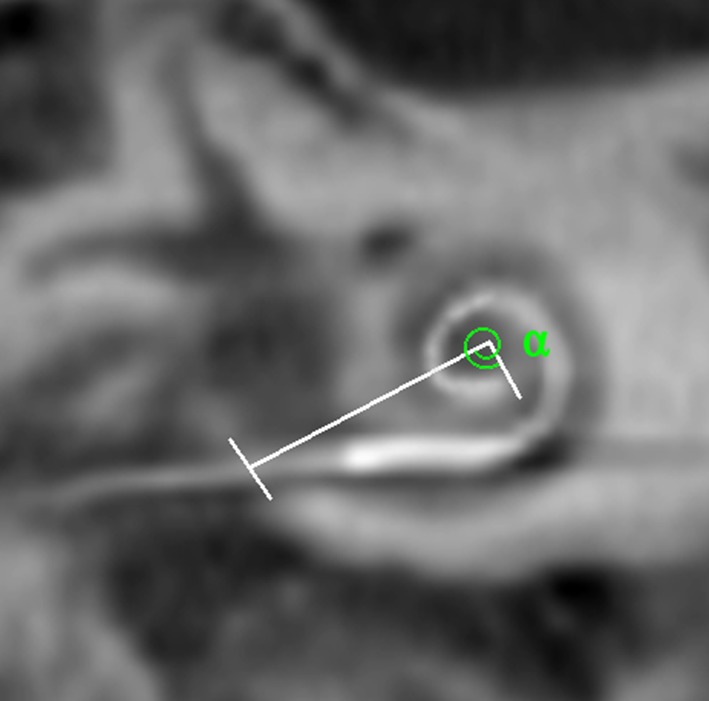
Method for the insertion depth angle (IDA) measurement. Starting point for the first line is  the level of RWM in middle of electrode, reaching to modiolus. Second line of the angle is drawn from modiolus to tip of electrode

On the first post-operative day, a cone-beam computed tomography (CBCT) was taken to assess the insertion results. The insertion depth angle (IDA) was measured and the scalar placement was evaluated (Fig. 1). All patients were discharged from the hospital on the first post-operative day. The patients did not receive any postoperative corticosteroid and/or antibiotic therapy.

The first postoperative hearing thresholds were mostly measured at the time of switch-on of the sound processor. Thresholds were measured routinely in the follow-up visits at approximately 6 and 12 months after activation.

The hearing preservation results are presented according to the following classifications used in the literature: The hearing thresholds were analyzed for PTA _(0.125–0.5 kHz)_, PTA _(0.125–1 kHz)_ and for HEARRING classification (*S*) [*S *= $1 - \left( {\frac{{{\text{PTApost}} - {\text{PTApre}}}}{{{\text{PTAmax}} - {\text{PTApre}}}}} \right)*100\%$] as described by Skarzynski et al. [30]. In the HEARRING classification, complete preservation was achieved whenever *S* > 75%, partial *S* = 75 − 25% and loss when <*S* 25%. For PTA _(0.125-0.5 kHz)_ and PTA _(0.125−1 kHz),_ complete hearing preservation was achieved when the mean pre- and postoperative threshold deterioration was ≤ 15 dB (HL) and partial hearing preservation when the threshold shift was ≤ 30 dB (HL). A postoperative threshold deterioration > 30 dB (HL) was classified as minimal preservation.

Data were analyzed with Statistical Packages for the Social Sciences (SPSS) for Windows version 25 (SPSS Inc., Chigaco, IL, USA). Wilcoxon signed rank test was used in the statistical analysis when comparing hearing results. The Pearson test was used as a correlation test.

## Results

All eighteen insertions could be performed through the RWM without any need to drill an extension. All insertions were carried out slowly, over 1 min. There were no post-operative complications. In the post-operative CBCT, the mean IDA was 395 degrees (range 313°–434°). All electrodes were fully inserted without any tip fold-over or scala translocations. The SME was located in close proximity to the modiolus in all but one ear. Information regarding the patient demographics and insertion results is summarized in Table 1. One patient with a psychiatric disorder (No. 13) insisted on the removal of the device after 3 months. Removal of cochlear implant was done 356 days after implantation. This patient has been excluded from end point results because data from a longer follow-up was not available.

**Table 1:** Patient demographics and insertion results

	Gender	Etiology	Age	Side	Approach	IDA	Electrode placement
1	Male	Mb Meniere	66	Right	RW	313	ST
2	Male	Usher Syndrome	25	Left	RW	412	ST
3	Female	Mb Meniere	41	Right	RW	405	ST
4	Male	SNHL	11	Right	RW	406	ST
5	Male	SNHL	17	Right	RW	396	ST
6	Female	SNHL	22	Right	RW	424	ST
7	Female	Usher Syndrome	11	Right	RW	390	ST
8	Female*	SNHL	45	Right	RW	434	ST
9	Female*	SNHL	45	Left	RW	410	ST
10	Female	Usher Syndrome	31	Right	RW	423	ST
11	Male	Usher Syndrome	28	Right	RW	392	ST
12	Female	SNHL	52	Left	RW	390	ST
13**	Male	Usher Syndrome	49	Right	RW	400	ST
14	Male	SNHL	67	Right	RW	400	ST
15	Female	SNHL	65	Right	RW	380	ST
16	Female	SNHL	71	Left	RW	360	ST
17	Male	SNHL	52	Right	RW	377	ST
18	Male	SNHL	24	Right	RW	391	ST
Mean			40			395	

*SNHL* progressive sensorineural hearing loss of unknown origin, *RW* round window, *IDA* insertion depth angle, *ST* scala tympani

*Bilateral implantee; **Explantation after 356 days due to maladaptation

The mean time between surgery and the first postoperative threshold measurements was 43 days (range 3–93, median 31). The mean follow-up time for all ears was 582 days (range 229–1041, median 482).

**Fig. 2:**
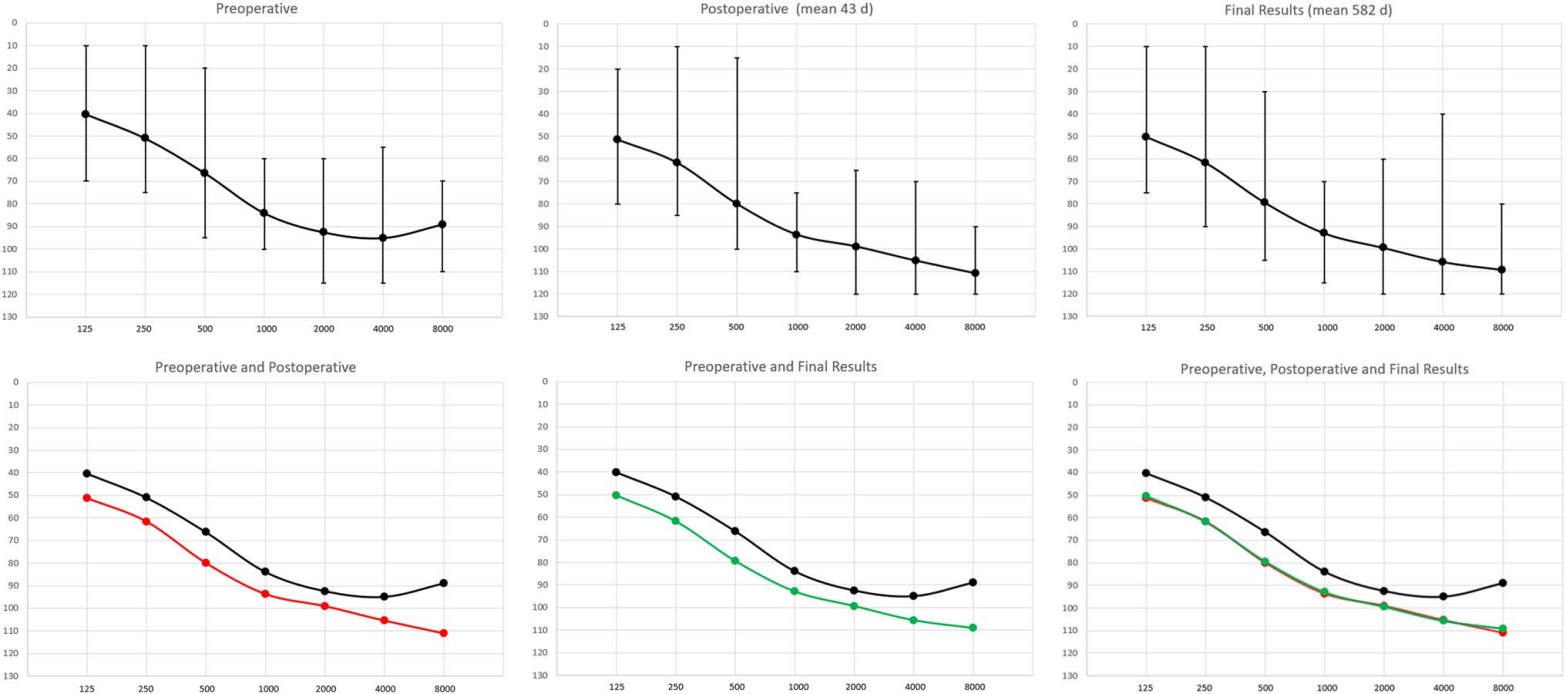
Mean hearing thresholds with minimum and maximum. a) preoperative b) early postoperative c) final follow-up

There were no total hearing losses. Functional low frequency hearing (PTA _(0.125−0.5 kHz)_ ≤ 80 dB (HL)) was preserved in 14 out of 17 ears (82%). The mean postoperative deterioration in the PTA _(0.125–0.5 kHz)_ was 11 dB (HL). At end of the follow-up, complete hearing preservation was achieved in 14 out of 17 ears (82.4%) for PTA _(0.125–0.5 kHz_ and in 13 out of 17 ears (76.5%)_)_ for PTA _(0.125–1 kHz)_. Partial preservation was achieved in 1/17 (5.8%) and 3/17 (17.6%) and minimal preservation occurred in 2/17 (11.8%) for PTA _(0.125–0.5 kHz__and in 1/17 (5.8%))_ for PTA _(0.125–1 kHz)_. The corresponding rates for earlier threshold measurements at 3–93 days after surgery, showed a complete preservation rate in 15 out of 18 ears (80.3%) for both PTA _(0.125–0.5 kHz)_ and PTA _(0.125–1 kHz)_. According to the HEARRING classification, 7 out of 17 ears (41%) had complete hearing preservation and 10 out of 17 ears (59%) had partial preservation at the end of follow-up. According to the earlier threshold measurements, complete hearing preservation was present in 55% of the ears and partial preservation in the remaining 45% when applying the HEARRING classification. The hearing preservation results according to the different classifications conducted in the early postoperative period and the final follow-up are summarized in Table 2a and b. The overall hearing results are illustrated in Fig. 2.

**Table 2:** Summary of hearing preservation results according to PTA 125–500 Hz, PTA 125–1000 Hz and HEARRING criteria described by Skarzynski et al. [30]. **a** Early postoperative. **b** Final follow-up

A											
Ears	Preop PTA_125–500 Hz dB (HL)_	Postop PTA_125–500 Hz dB (HL)_	Δ PTA _125–500 Hz dB (HL)_	Hearing preservation	Preop PTA _0.125–1 kHz dB (HL)_	Postop PTA _0.125–1 kHz dB (HL)_	Δ PTA_0.125–1 kHz dB (HL)_	Hearing preservation	Hearring (%)	Hearring	Postoperative audiogram (d)
1	48	60	12	Complete	51	64	13	Complete	94	Complete	93
2	73	87	14	Complete	78	90	12	Complete	55	Partial	41
3	60	73	13	Complete	61	76	15	Complete	90	Complete	3
4	65	80	15	Complete	69	84	15	Complete	64	Partial	62
5	52	62	10	Complete	61	71	10	Complete	83	Complete	48
6	73	83	10	Complete	80	90	10	Complete	55	Partial	27
7	77	87	10	Complete	78	89	11	Complete	58	Partial	89
8*	40	53	13	Complete	49	63	14	Complete	69	Partial	57
9*	42	53	11	Complete	56	64	8	Complete	90	Complete	83
10	73	75	2	Complete	80	83	3	Complete	82	Complete	31
11	68	75	7	Complete	73	80	7	Complete	79	Complete	37
12	13	15	2	Complete	31	33	2	Complete	98	Complete	30
13	43	77	34	Minimal	56	83	27	Partial	47	Partial	29
14	33	42	9	Complete	44	51	7	Complete	92	Complete	28
15	27	43	16	Partial	39	56	17	Partial	78	Complete	27
16	57	62	5	Complete	59	65	6	Complete	88	Complete	28
17	70	80	10	Complete	71	84	13	Complete	66	Partial	30
18	28	45	17	Partial	39	56	17	Partial	74	Partial	30
Mean	52	64	12	15/18	60	71	12	15/18	76	10/18	43

B											
Ears	Preop PTA _125–500 Hz dB (HL)_	Final PTA _125–500 Hz dB (HL)_	Δ PTA _125–500 Hz dB (HL)_	Hearing preservation	PTA _0.125–1 kHz dB (HL)_	Final PTA _0.125–1 kHz dB (HL)_	Δ PTA _0.125–1 kHz dB (HL)_	Hearing preservation	Hearring (%)	Hearring	Follow-up (d)
1	48	87	39	Minimal	51	90	39	Minimal	29	Partial	779
2	73	73	0	Complete	78	79	1	Complete	55	Partial	434
3	60	62	2	Complete	61	64	3	Complete	95	Complete	854
4	65	67	2	Complete	69	70	1	Complete	64	Partial	436
5	52	60	8	Complete	61	70	9	Complete	87	Complete	784
6	73	87	14	Complete	80	93	13	Complete	42	Partial	587
7	77	85	8	Complete	78	88	10	Complete	63	Partial	482
8*	40	55	15	Complete	49	64	15	Complete	69	Partial	229
9*	42	50	8	Complete	56	66	10	Complete	69	Partial	363
10	73	78	5	Complete	80	85	5	Complete	90	Complete	660
11	68	73	5	Complete	73	78	4	Complete	74	Partial	608
12	13	17	3	Complete	31	35	4	Complete	95	Complete	388
13**	43	n/a	n/a	n/a	56	n/a	n/a	n/a	n/a	n/a	29
14	33	67	34	Minimal	44	70	26	Partial	62	Partial	969
15	27	50	23	Partial	39	65	26	Partial	63	Partial	462
16	57	65	8	Complete	59	69	10	Complete	76	Complete	403
17	70	77	7	Complete	71	79	24	Partial	97	Complete	1041
18	28	33	5	Complete	39	46	8	Complete	90	Complete	413
Mean	52	64	11	14/17	60	71	12	13/17	72	7/17	582

*Bilateral implantee; Hearing preservation: Complete Δ PTA ≤ 15 dB (HL); Partial Δ PTA ≤ 30 dB (HL); Minimal Δ PTA > 30 dB (HL) but reliably measurable

*Bilateral implantee; **Explantation after 356 days due to maladaptation; Hearing preservation: Complete Δ PTA ≤ 15 dB (HL); Partial Δ PTA ≤ 30 dB (HL); Minimal Δ PTA > 30 dB (HL) but reliably measurable

We found a moderate correlation between the patient’s age and the deterioration of the residual hearing at the final follow-up. The correlation coefficients were *r* = 0.603 (*p* = 0.01) for PTA _(0.125–0.5 kHz)_ and *r* = 0.613 (*p* = 0.009) for PTA _(0.125−1 kHz)_. There was no correlation between the baseline hearing and the preservation after surgery. For PTA _(0.125–0.5 kHz)_, the correlation coefficient was *r* = − 0.341 (*p* = 0.180); for PTA _(0.125−1 kHz)_ the value of *r* was − 0.417 (*p* = 0.096). We did not detect any significant differences between etiologies and the deterioration of residual hearing at the end of follow-up (PTA_125−500 Hz_*p* = 0.768 and for PTA_125−1000 Hz_*p* = 0.649).

There were 14 ears (78%) eligible for possible EAS and the vast majority, i.e. 13 ears (72%) were primarily fitted with EAS. Subsequently, eight patients (nine ears) continued to use EAS. Two patients did not experience any subjective benefit from simultaneous acoustic stimulation and three patients preferred an open ear canal to the EAS strategy. The patient with bilateral SMEs used an EAS strategy in both of her ears.

The mean preoperative SRT was − 1.2 dB (SNR) (range − 6.8 to + 10.0 dB (SNR)). The postoperative SRT improved significantly and was − 5.2 dB (SNR) (range − 8.5 to − 0.7 dB (SNR)). The improvement of Δ − 4.0 dB (SNR) with the Finnish matrix sentence was statistically significant (*p* = 0.01) and in the clinically expected magnitude. We found a significant (negative) correlation between IDA and post-operative speech test results (*r* = − 0.617; *p* = 0.014), i.e. better post-operative speech test results with deeper IDA. No correlations were detected for the pre- and postoperative SRT values (*r* = 0.251; *p* = 0.367).

## Discussion

The SME was originally developed to achieve less traumatic insertions through either the round window or via a cochleostomy. Our pre-clinical study revealed very consistent insertion results and one scala translocation out of twenty insertions in fresh frozen temporal bones [31]. Although the SME was not originally designed for hearing preservation, we observed good preservation of the residual hearing in our first clinical patients. Encouraged by these results, we started to use the SME also in patients with better hearing thresholds and ultimately even in patients eligible for EAS fitting. This study describes the SME’s clinical results with an emphasis on hearing preservation in 17 consecutive patients (18 ears) with meaningful residual hearing.

Similar to the temporal bone study, the overall surgical handling was reasonably good. However, in patients with a narrow facial recess, the visibility to the round window may be obstructed by the bulky array-sheath assembly and in two cases, this compelled us to switch devices in favor of a slim LWE. Aschendorff et al. [32] also reported difficulties in the overall access to the round window area in some cases. Impaired visibility may easily lead to surgical inadequacies or even errors. Another surgical issue is that the inferior lip of the silicone sheath occasionally becomes stuck at the inferior border of the crista fenestra, complicating the introduction of the sheath into the cochlea. Upon loading of the array, the tip of the silicone sheath may open and spread which aggravates the aforementioned issue. Cuda and Murri [33] reported problems in two out of 61 insertions; in these two cases, several reloads and insertion attempts were required to achieve adequate insertion.

The insertion results with SME appear to be rather consistent. All insertions were performed through the RWM without any need for drilling an inferior extension. The mean IDA in our clinical series was 395°, which is almost identical to the IDA found in a temporal bone study and also similar to that reported in other studies [31, 32, 34]. Therefore, the cochlear coverage appears to be adequate for pure electrical stimulation.

Current publications have reported significantly higher rates of tip fold-over for the SME (4.5–7.7%), compared to other LWE’s (approx. 1%) or stylet-type PME’s (approx. 2–3%) [32, 35–39]. McJunkin et al. [35] reported about 9 tip fold-overs out of 117 insertions (7.7%), Gomes et al. [37] about two out 40 insertions (5%) and Friedmann et al. [36] about 11 out of 237 insertions (4.6%). In a multicenter study, Aschendorff et al. [32] reported two tip fold-overs out of 44 insertions (4.5%), which they attributed to surgical error. Unfortunately, they did not provide any detailed description of the specific error other than noting that the surgeon was not sufficiently experienced. Nevertheless, these reports demonstrate that postoperative imaging and/or specific electrophysiological measurements are necessary to exclude tip fold-over with this array.

In our cohort of patients, we found no scala translocation on the CBCT images and all electrodes were in the scala tympani. McJunkin et al. [36] described scala translocation in 3 out of 23 insertions (13%), whereas Aschendorff et al. [32] reported of no scala translocations. In summary, the translocation rates of the SME appear to be considerably lower than those reported for stylet-type PMEs, in the publications, their translocation rates have varied from 15.8% up to 52.3% [1, 2, 16, 22, 40, 41].

There are many different classifications for defining postoperative hearing preservation. We chose to present our data according to the most common definitions used in the literature. The hearing preservation rates achieved with the SME appear to be superior to other stylet-type PMEs [21, 42, 43]. The majority of patients (72%) were initially fitted with an EAS strategy and 44% experienced benefits with the acoustic stimulation and continued to use the device. Roland et al. [11] reported on 50 patients eligible for EAS who were implanted with short 16 mm LWE; of these, 33 (66%) were postoperatively fitted with an EAS processor and 23 patients were still benefiting from EAS 5 years after surgery [44]. Although the overall hearing preservation results of the SME appear to be inferior to those reported for a shorter 16 mm LWE, the SME has the clear advantage of providing adequate cochlear coverage for pure electric stimulation should the residual hearing deteriorate. Therefore, it eliminates the possible need for re-implantation with a longer electrode. Roland et al. [11] reported the need for five revision surgeries out of 50 EAS patients (10%) in which the 16 mm LWE had to be replaced with a longer array to provide adequate hearing performance with electric stimulation. In our study, the mean postoperative threshold deterioration for PTA _(0.125–1 kHz)_ was 12 dB (HL), which is comparable to the value reported by Gantz et al. [7], who found a mean threshold decline of 9 dB (HL) with a 16 mm LWE. Ramos et al. [43] compared the hearing preservation results of the SME with a 20 mm slim LWE and stylet-type PME. The hearing preservation (PTA _(0.125–0.750 kHz)_ < 15 dB (HL)) results with the SME (50%) and the slim LWE (43%) were similar to our series, whereas very poor hearing preservation (0%) was encountered with the stylet-type PMEs.

When comparing the results according to the HEARRING classification, complete preservation was observed in 47% of ears. In pediatric patients, Manjaly et al. [45] reported complete hearing preservation in 55% for 20 mm and 28 mm LWEs. There is another report of complete hearing preservation in nine out 25 patients (35%) with LWEs of different lengths after 1 year [46].

LWEs are reported to achieve low frequency hearing preservation in a wide range from 11.3 to 77.7% [14, 17, 18, 20]. Our results were 82.4% for PTA _(0.125−0.5 kHz)_ and 76.5% for PTA _(0.125−1 kHz)_. Although there is some variation in the methods of assessing low-frequency preservation between different studies, we have achieved comparable short-term preservation results with the SME as reported for LWE. We were not able to measure the thresholds immediately after surgery, which raises the question of whether the threshold shift was due to direct insertion trauma or to postoperative inflammation.

We found an age-related effect on the postoperative hearing preservation. Zanetti et al. [47] reported better hearing preservation rates with children as compared to adults, but this difference was not statistically significant. In the systematic review conducted by Causon et al. [48], age was not a significant factor for hearing preservation. Thus, it is uncertain whether age is a contributing factor behind hearing preservation. We found no correlation between the baseline residual hearing with the preservation rates.

We found a significant improvement in the speech recognition in noise as measured with the FMST. The mean improvement of the speech reception threshold in noise after implantation of 4.0 dB (SRN) was clinically most significant and in the range of the desired and expected improvement [29]. One interesting finding was that the postoperative SRT correlated significantly with the insertion depth (i.e. the deeper the insertion, the better the postoperative SRT). There is published data which has revealed a correlation between deeper insertion angles with better postoperative hearing outcomes [4, 15, 40]. This correlation is all the more surprising, since in our series there were seven patients (eight ears) with EAS for whom the IDA is not considered to be critical. However, caution is necessary in the interpretation of this correlation due to the small number of patients.

The limitations of this study are inherent in its retrospective nature. The statistical power of the analysis is weakened by the small size and heterogeneity of the cohort. Additionally, our finding should be regarded as preliminary, since a longer follow-up will be needed to evaluate the long-term hearing preservation results.

## Conclusion

The hearing preservation results with the SME were superior to those reported for stylet type PMEs. In several cases, residual hearing was well preserved which enabled patients to use EAS stimulation. Although the hearing preservation rate of the SME was inferior to that achieved with short LWEs, it provided deeper insertions and better cochlear coverage for pure electrical stimulation in the event of post-operative or progressive hearing loss. This may have obviated the need for re-implantation with a longer electrode in the event of postoperative or progressive hearing loss.
